# Age of European silver eels during a period of declining abundance in Norway

**DOI:** 10.1002/ece3.6234

**Published:** 2020-04-12

**Authors:** Caroline M. F. Durif, Ola H. Diserud, Odd Terje Sandlund, Eva B. Thorstad, Russell Poole, Knut Bergesen, Rosa H. Escobar‐Lux, Steven Shema, Leif Asbjørn Vøllestad

**Affiliations:** ^1^ Institute of Marine Research Austevoll Research Station Storebø Norway; ^2^ Norwegian Institute for Nature Research Trondheim Norway; ^3^ Marine Institute Newport Co. Mayo Ireland; ^4^ Norwegian Institute for Nature Research Ims Research Station Sandnes Norway; ^5^ Grótti ehf. Reykjavík Iceland; ^6^ Department of Biosciences Centre for Ecological and Evolutionary Synthesis University of Oslo Oslo Norway

**Keywords:** aging method, *Anguilla anguilla*, catchment, endangered species, growth, migration, otolith, river, sex ratio

## Abstract

The European eel (*Anguilla anguilla*) is critically endangered throughout its range. Knowledge about age distribution of future spawners (silver eels) is essential to monitor the status and contribute to the recovery of this species. Determination of age in anguillid eels is challenging, especially in eels from the northern part of the distribution area where growth is slow and age at maturation can be up to 30 years or more. Eels from the river Imsa in Norway have been monitored since 1975, and this reference time series has been used to assess the stock at the European level. Population dynamics in this catchment were analyzed during the late 1980s by estimating ages on whole cleared otoliths. However, techniques for revealing annual increments on otoliths have evolved over the years sometimes yielding significant differences in age estimates. In this study, the historical otolith data were reanalyzed using a grinding and polishing method rather than reading the whole otolith. The new age estimates were considerably higher than the previous ones, sometimes by up to 29 years. Since the 1980s, mean age of silver eels only slightly increased (from 19 to 21 years in the 2010s). This was mainly due to the disappearance of younger silver eels (<15 years) in the 2010s. The new age estimates agreed with the steep decline in recruitment which occurred in the late 1980s in the Imsa catchment. Mean growth (30 mm/year, min–max: 16–64 mm/year) has not changed since the 1980s, although density in the catchment has decreased. Revealing and reading age of slow‐growing eels remain a challenge but adding a measure of otolith reading uncertainty may improve age data collection and contribute to recovery measures for this species.

## Graphical abstract

We reanalyzed a historical European eel otolith collection using more recent aging techniques. New estimates of age at maturation were up to 30 years higher than previously thought. Since the decline of eels in the late 1980s, mean age of silver eels has only slightly increased, from 19 to 21 years in the 2010s. Growth (30 mm/year) has not changed since the 1980s, although eel density in the catchment has decreased.

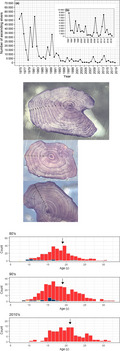

## 1 INTRODUCTION

Despite their remarkable ability to adapt to all kinds of environments, the European eel, *Anguilla anguilla,* population has been in decline, at least since the 1960s, and probably even since the early 1900s coinciding with the reduction of freshwater habitats and developing eel fishing industry (Dekker, 2019; Dekker & Beaulaton, 2016; ICES, 2019). Recruitment to freshwater habitats decreased by more than 90% in the early 1980s, and since 2008, the European eel has been listed as critically endangered on the International Union for Conservation of Nature (IUCN) red list (Jacoby & Gollock, 2014). Causes for the decline are related to habitat loss, hydropower, overfishing, climate change, pollution, parasites, and diseases (Aschonitis et al., 2017; Castonguay & Durif, 2016; Drouineau et al., 2018).

The European eel is semelparous and panmictic (Als et al., 2011). It spawns in the Sargasso Sea, but is distributed across Europe, from northern Norway to northern Africa and far into the Mediterranean (Dekker, 2003a; Schmidt & Regan, 1923). Larvae drifting with the Gulf Stream metamorphose into glass eels when they reach the continental shelf. These glass eels colonize coastal and freshwater habitats where they spend their growth phase before maturing into silver eels which will migrate back to the Sargasso Sea for spawning (Bertin, 1956; Tesch, 2003).

The status of the stock is primarily assessed through time series of recruiting glass eels (or elvers which are pigmented 0+ age eels) to freshwater at different monitoring stations across Europe (ICES, 2000). A severe reduction in glass eel recruitment, more marked in the northern part of the distribution area, became apparent in the early 1980s (Bornarel et al., 2018; Dekker, 2003b, 2004; ICES, 2016; Moriarty, 1986, 1990). However, signs of decrease in the standing stock (yellow stage) date from the 1960s (Aalto et al., 2016; Dekker, 2003c).

At some of the monitoring stations, like in the river Imsa, Norway, both upstream ascending elvers and downstream migrating silver eels are trapped and counted (Sandlund et al., 2017). The Imsa reference time series was started in the 1970s, and the age distribution and the population dynamics in this catchment were especially studied in the 1980s and 1990s, yielding fundamental knowledge on the ecology of European eels (Vøllestad & Jonsson, 1986, 1988; Vøllestad, Jonsson, Hvidsten, & Næsje, 1994; Vøllestad et al., 1986). Following the awareness of the European eel population crash, countries across Europe developed management plans in accordance with a European Union Regulation (EU, 2007). Although a non‐EU country, Norway followed in 2010, with a ban on eel fishing and the Imsa time series became even more relevant to monitor the local sub‐stock (Poole et al., 2018).

Knowledge on age structure is essential to assess the status of a population, in terms of recruitment limitations or overfishing, and it can provide feedback on the effectiveness of management practices (Hilborn & Walters, 1992; Quist, Pegg, & DeVries, 2012). Determination of age in fish is challenging, especially in long‐lived species such as the European eel (Kullmann et al., 2018; Poole & Reynolds, 1996; Vøllestad, Lecomte‐Finiger, & Steinmetz, 1988). For this species, otoliths (or earstones) are prepared so that annual rings (annuli), marking periods of fast and slow growth, become visible and can be counted to give an age estimate (Moriarty, 1973, 1983; Svedäng, Wickström, Reizenstein, Holmgren, & Florenius, 1998). Four main methods have been used for preparing otoliths: (a) grinding and polishing, (b) slicing, (c) burning and cracking, and (d) clearing of whole otoliths in ethanol (in toto method). It has been debated what is the better method (ICES, 2009; Vøllestad & Næsje, 1988). Different methods yield different estimates (Moriarty & Steinmetz, 1979). The suitability of each method depends on the age and growth of individuals (Vøllestad et al., 1988). For example, burning and cracking is more suitable for slow‐growing eels, because of the shape of their otolith and the numerous but narrow growth increments (Vøllestad & Næsje, 1988). The “in toto” method (clearing whole otoliths) is fast and inexpensive, but best suited for young eels (Vøllestad & Næsje, 1988). A manual regarding the best practice for aging eels has been developed (ICES, 2009; 2011). However, there is still some debate due to a general lack of validation and of different growth patterns in such a widespread species.

Once annuli are revealed, interpretation remains challenging. For example, traumatic events, such as high temperatures in the summer, diseases, or stress can cause supernumerary rings or “false” checks (Deelder, 1976; Domingos, Costa, & Costa, 2006; Graynoth, 1999; ICES, 2009; Svedäng et al., 1998; Tzeng, Wu, & Wickstrom, 1994). Translocation of young eels for stocking measures can also cause stress marks, which can be misinterpreted as winter annuli (Kullmann et al., 2018). In addition, observations can vary between readers, over time and between laboratories.

Age estimates have been validated in some cases using chemical marking of otoliths (Chrisnall & Kalish 1993; Dekker, 1986; Oliveira, 1996; Svedäng et al., 1998), external color marking (Chisnall & Kalish, 1993; Poole & Reynolds, 1996) or indirect methods using individual mark‐recapture techniques (Beentjes & Jellyman, 2015; Poole & Reynolds, 1996), or by introducing eels in a pristine waterbody (ICES, 2009; Vøllestad & Næsje, 1988; Wickström, Westin, & Clevestam, 1996). However, validations for the European eel have been done mostly on young individuals with a maximum age of 14 years, and unfortunately, there are still too few examples of validation of aging methods in eels.

In the earlier years (1980–1990s), the age of silver eels from the river Imsa was determined using the in toto method (IT), and it was later suspected that these ages were underestimated. Since the otoliths had been maintained, it was decided to reanalyze them using the consensus method: grinding and polishing method (GP) and to compare both age estimates. Additionally, new otolith samples were collected in the 2010s and treated in the same way (GP method) to investigate possible changes in the age distribution of silver eels since the decline of the population (Poole et al., 2018). Finally, using this new dataset, the possible relationship between the number of ascending recruits and the number of descending silver eels was examined to reanalyze previous models (Vøllestad & Jonsson, 1988) established for the Imsa eel stock during the period 1975–1987.

## 2 MATERIAL AND METHODS

### 2.1 Study location

**FIGURE 1:**
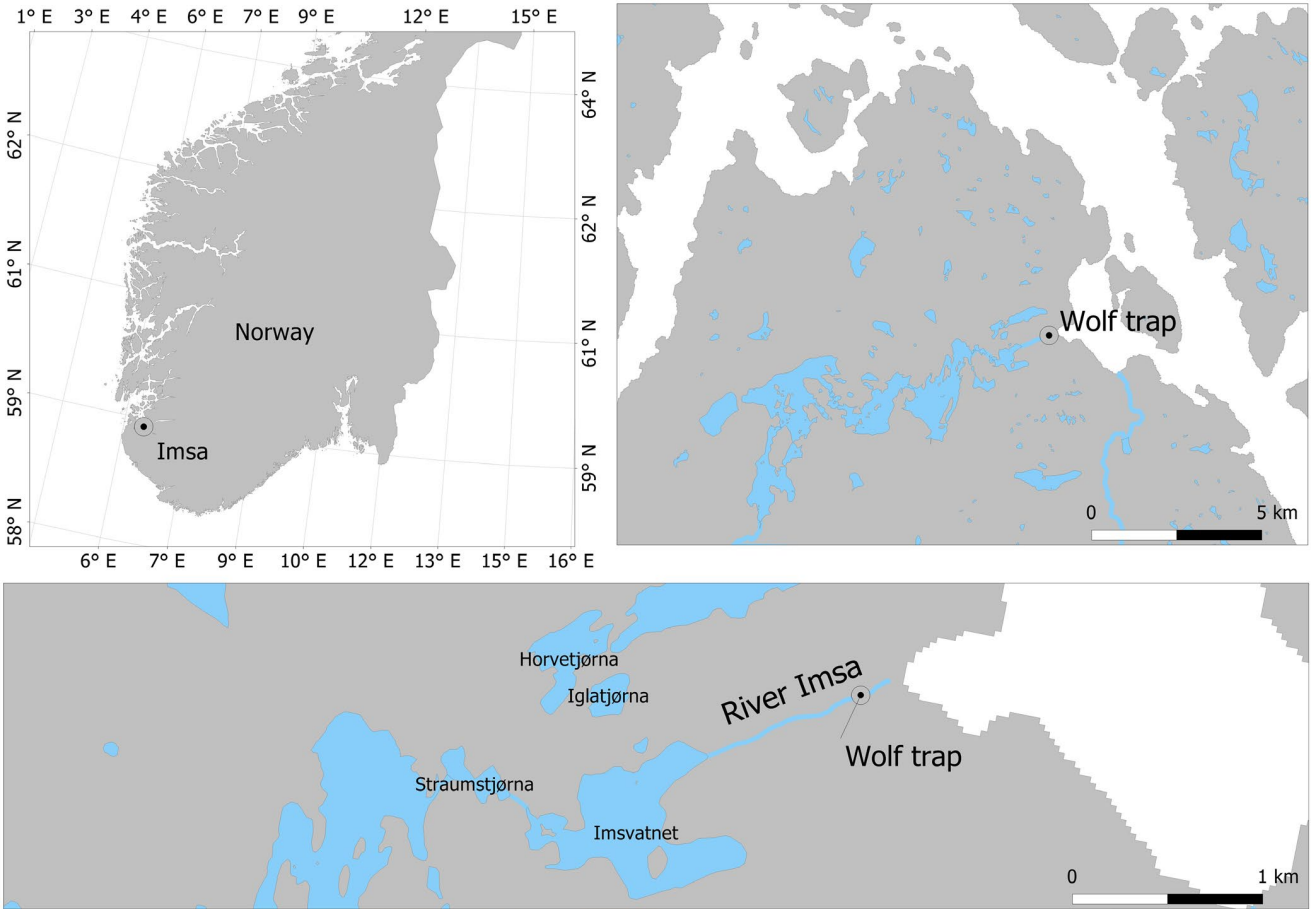
Map of the study area showing the location of the river Imsa and of the trap capturing out migrating silver eels (*Anguilla anguilla*)

The river Imsa in southwestern Norway (Figure 1) is an unregulated oligotrophic system. The catchment covers an area of 12,800 ha, of which 1,536 ha (12%) is lake surface (major lakes are Imsvatnet, 40 ha, and Storavatnet, 819 ha). The river Imsa has been part of a large experimental research station since 1975.

Traps catching all descending silver eels as well as all ascending juveniles were established in 1975 and have been in continuous operation since they were established. The traps are located about 100 m upstream from the river outlet in the sea. Both traps are checked at least twice every day (at circa 08:00 and 15:00).

The distance from the traps to the upper end of eel habitat in the catchment is around 20 km, and the eels ascend the system up to an altitude of ~215 m above sea level (Vøllestad & Jonsson, 1986, 1988). The river support populations of anadromous Atlantic salmon *Salmo salar* and brown trout *S. trutta*, and the upstream lakes support populations of brown trout, Arctic charr *Salvelinus alpinus,* whitefish *Coregonus lavaretus,* and threespine stickleback *Gasterosteus aculeatus*.

Descending, predominantly silver eels are caught in a Wolf trap (mesh size 10 mm, inclination 1:10). Wolf traps generally catch all eels larger than ~25 cm in length, which includes all silver eels in the river Imsa (Vøllestad & Jonsson, 1986). This trap is collecting all downstream migrating fish and is in operation all year round. The trap is functional at all water levels.

The juveniles entering this watershed are small yellow eels (elvers or recruits) that are typically 70–90 mm long and weighing less than 1.0 g, although a few individuals may be larger. The elver trap leads all ascending recruits into a capture chamber where their numbers are recorded, and sub‐sampling of size is performed, before they are released to continue upstream.

**FIGURE 2:**
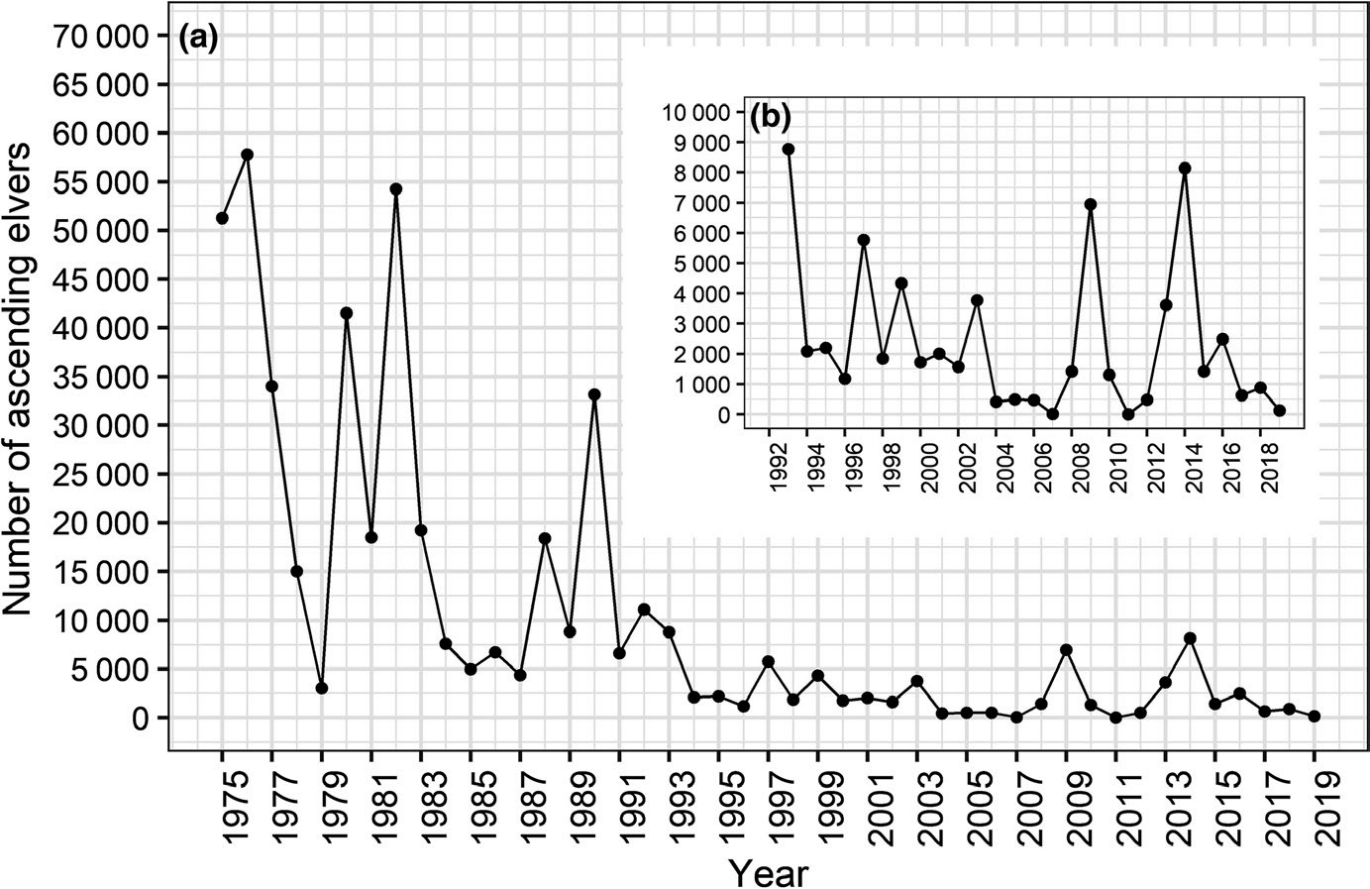
Annual number of European eels recorded in the traps in the river Imsa, 1975–2019. (a) upstream migrating recruits (mostly young of the year elvers) in the spring; (b) zooms on the period 1992–2019 and downstream migrating silver eels in the fall

**FIGURE 3:**
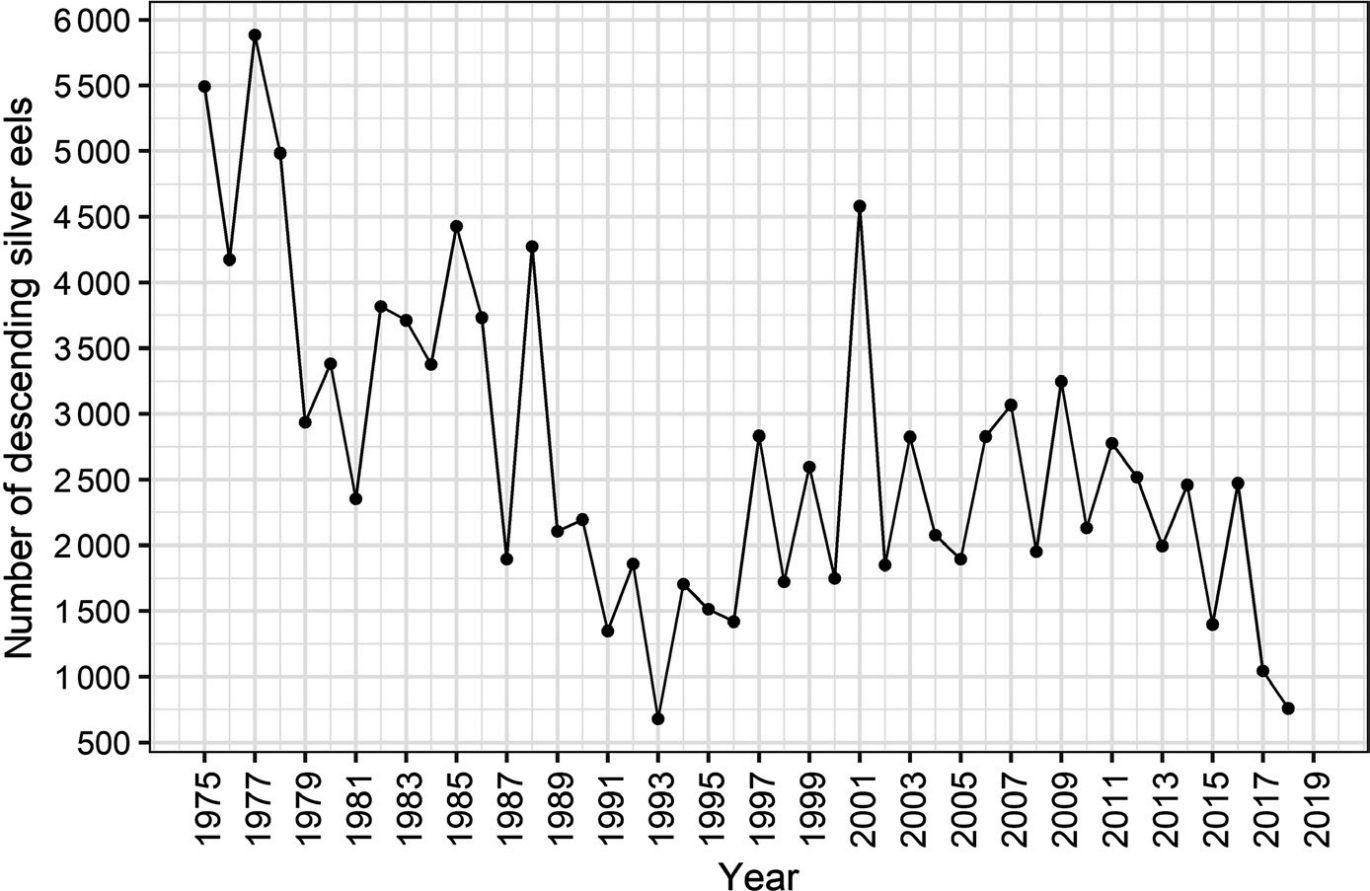
Annual number of downstream migrating silver European eels in the fall recorded in the traps in the river Imsa, 1975–2019

The distance from the nearest lake along the free‐flowing river to the fish trap is 970 m. There has been no stocking of eels in this watershed. Before 2006, there was a restricted seasonal yellow and silver eel fishery upstream of the trapping station. The number of ascending recruits and descending silver eels is given per calendar year (Figures 2 and 3).

### 2.2 Otolith data and age determination

Otoliths from the historical collection (collected between 1982 and 1992) were initially read in toto by clearing them in 96% ethanol for 18–24 hr before counting the annuli directly using a stereo microscope and 96% ethanol as refraction medium (Vøllestad, 1985). These otoliths were, since then, stored dry in an envelope, each labeled with length, sex, and stage (yellow or silver). For the reanalysis, a subsample was randomly selected from years with the highest sampling effort (1982:224 otoliths, 1986:102 otoliths, 1991:219 otoliths, and 1992:117 otoliths).

The more recent otoliths (from the 2010s) were sampled from eels caught during their downstream run in the river Imsa. Twenty‐five silver eels were sacrificed per year (61 eels in 2016). Length, weight, fin, and eye diameters were measured for stage determination (Durif, Dufour, & Elie, 2005; Durif, Guibert, & Elie, 2009). The eels were dissected for sex determination and removal of otoliths. Otoliths from the 2010s were not analyzed using the old in toto method.

**FIGURE 4:**
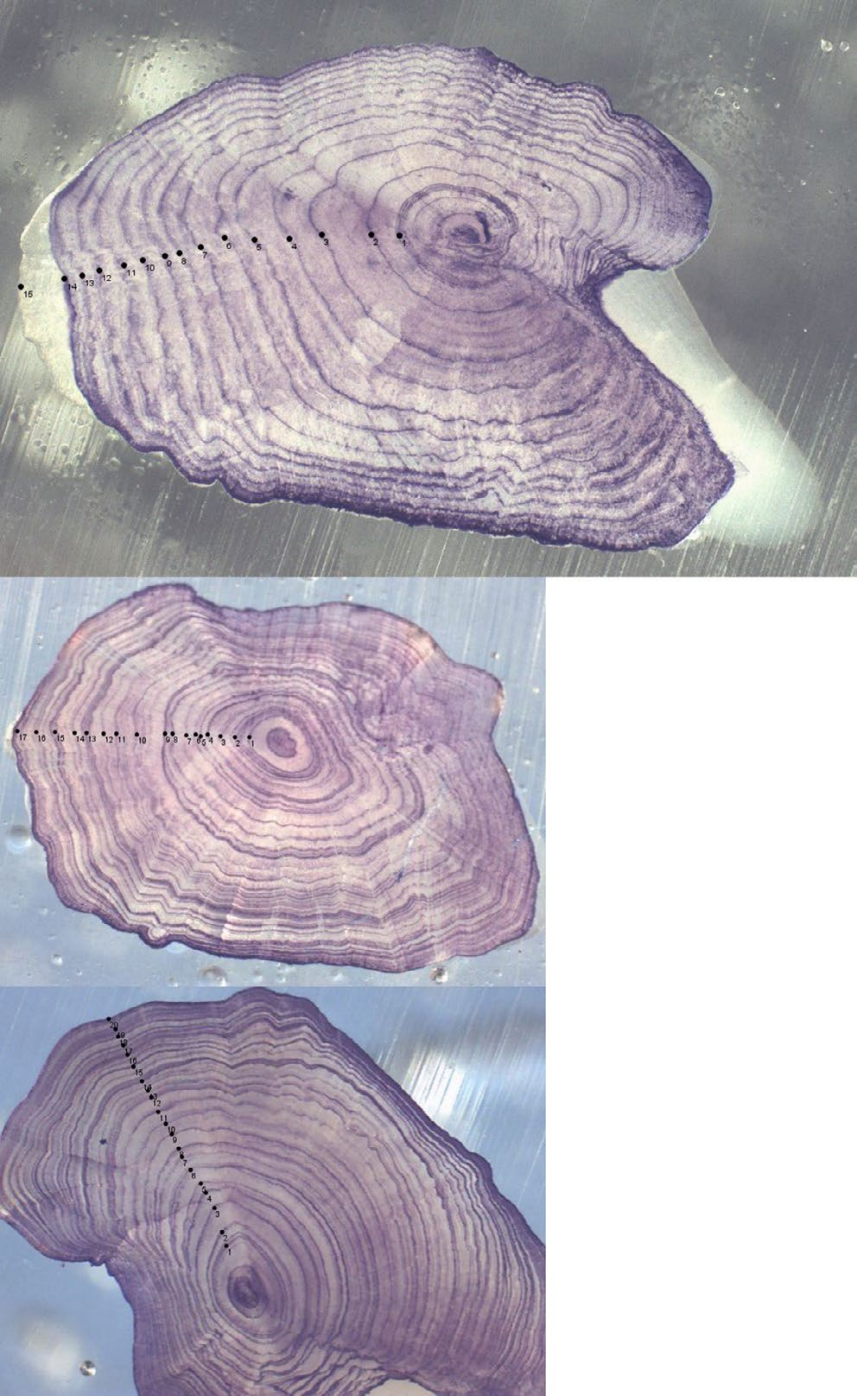
European eel otoliths after grinding, polishing, etching, and staining. Annual rings are numbered on the pictures. Each otolith was assigned an Otolith Uncertainty Index (OUI) which corresponds to differences in readings between observers, level 1: <3 years, level 2: 3–5 years, and level 3: more than 5 years. 2A: 15 years, OUI 1, body length: 67 cm, (In toto estimate: 9 years). 2B: 18 years, OUI 2, body length: 69 cm, (In toto estimate: 6 years). 2C: 20 years, OUI 3, body length: 67 cm, (In toto estimate: 9 years)

A total of 798 fish were processed. All otoliths (historical and new) were prepared by grinding, etching, and staining and read according to the protocol described in ICES, (2009, 2011; Figure 4). The year 0 band was assigned as the first winter after the oceanic migration, *that is,* it marked the beginning of the continental life stage. The last year was defined as the outer edge of the otolith since eels were caught during the fall season. Some otoliths had clear and regularly spaced annuli (Figure 4a). Others presented numerous tight rings, unevenly spaced, which sometimes joined in a “bundle” or fused into one large annulus on the other side of the otolith (Figure 4b, c). Whether these bundles represent one or several years is unknown. Here, we assumed that some of the marks forming a bundle represented false checks and thus one bundle represented one year. Otoliths were read by two or three observers, or for some samples by the same observer twice, but the second time several months after the first reading. As expected, some otoliths were easier to interpret than others, and the age estimates did not always agree between observers. To characterize the uncertainty in the readings, we assigned each age estimate with an Otolith Uncertainty Index (OUI) corresponding to how much the observations differed between observers/observations:

- OUI level 1: differences <3 years
- OUI level 2: differences between 3 and 5 years
- OUI level 3: differences of more than 5 years.

### 2.3 Calculations and statistics

Indicative growth rate was determined using body length at age of capture (LT). For each individual, it was calculated by dividing LT (mm) of the eel minus 70 mm, which is the mean size of glass eels when they recruit to European coasts (Elie, 1979; Svedäng, Neuman, & Wickström, 1996), by the continental age.

Differences in mean age and mean length between decades and between OUI levels were tested using linear regression models. Differences in proportion of OUI levels between decades were tested using a chi‐square test. Differences between length–weight relationships were tested with ANCOVA after the variables were log‐transformed to investigate changes in condition over the decades. Statistics were carried out using the statistical software R (R Core Team, 2019, v. 3.6.0).

## 3 RESULTS

### 3.1 Age estimates

Otoliths from 41 eels (5%) were unreadable and were excluded from the analyses and the following percentages (Table 1). Most otoliths (only GP readings) were assigned an OUI (Otolith Uncertainty Index) level 2, indicating a 4 to 5 years uncertainty (47%). OUI level 1 otoliths (1‐ to 3‐year uncertainty) represented 14%, and OUI level 3 (over 5‐year uncertainty) represented 39% of the otoliths analyzed. Age varied with OUI (*R* ^2^ = 0.04, *F*(2, 753) = 17.8, *p* < .0001): uncertainty increased with age. Length did not vary between OUI levels (*R* ^2^ = 0.006, *F*(2, 750) = 2.2, *p* = .11). The proportion of OUI level 3 otoliths was different across decades (*Χ* ^2^ = 45.729, *df* = 4; *p* < .0001): 51% in the 1980s, 28% in the 1990s, and 34% in the 2010s.

**Table 1:** Summary of the otolith sample collected from silver stage European eel caught in a Wolf trap from the river Imsa (Norway)

Year	Number of fish sampled	Number aged	Number not readable	Percent not readable	Age of females (mean ± *SD*; years)	Length of females (mean ± *SD*; cm)	Number of eels in OUI level 1, 2, and 3 respectively	Age of males (mean ± *SD*; years)	% of males
1980s	326	282	22	7	19 ± 4	60 ± 7	28/107/147	14 ± 4	8
1990s	336	304	12	4	19 ± 5	63 ± 11^b^	54/159/91	15 ± 3	5
2010s	136	129	7	5	21 ± 4^a^	68 ± 7^b^	11/74/44	none	0
SUM	798	757	41	5	19			15	5 (7 excluding the 2010s)

Notes

OUI: Otolith Uncertainty Index describing the level of uncertainty when otolith was read by different readers, level 1: 1–3 years, level 2: 4–5 years, and level 3: more than 5 years.

^a^ Mean age of females became significantly higher only in the 2010s. Using all otoliths: *R* ^2^ = 0.03, *F*(2, 712) = 10.23, *p* < .0001; *t* tests 1980s versus 1990s: *p* = .34 and 1980s versus 2010s: *p* < .0001. Removing OUI level 3 otoliths: *R* ^2^ = 0.05, *F*(2, 430) = 10.43, *p* < .0001; *t* tests 1980s versus 1990s: *p* = .10 and 1980s versus 2010s: *p* < .0001.

^b^ Mean length of females increased significantly between each decade, *R* ^2^ = 0.09, *F*(2, 744) = 35.73; *p* < .0001); *t* tests 1980s versus 1990s and 1980s versus 2010s: *p* < .0001.

**FIGURE 5:**
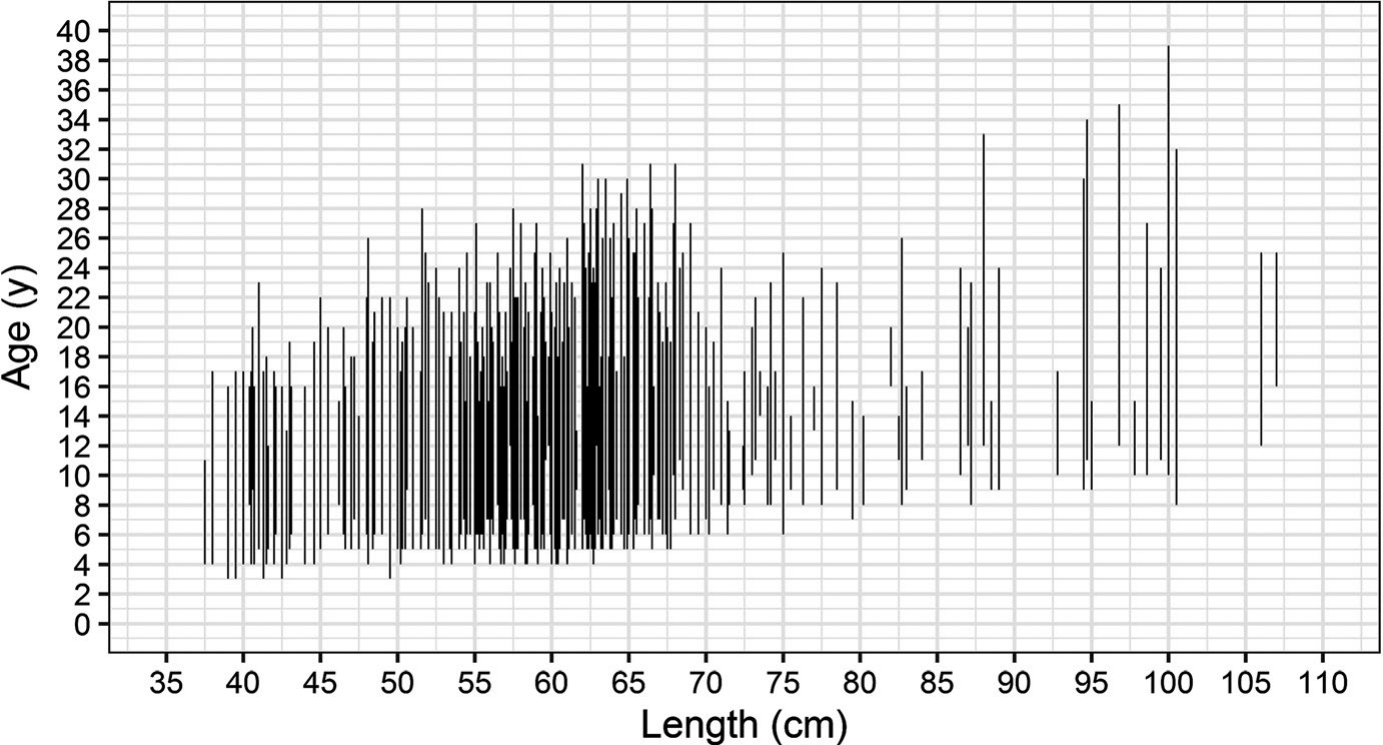
Differences in age estimates of eel (*Anguilla anguilla*) between two methods either by reading the otoliths in toto (lower end of the bar) or grinding and polishing (top end of the bar)

**FIGURE 6:**
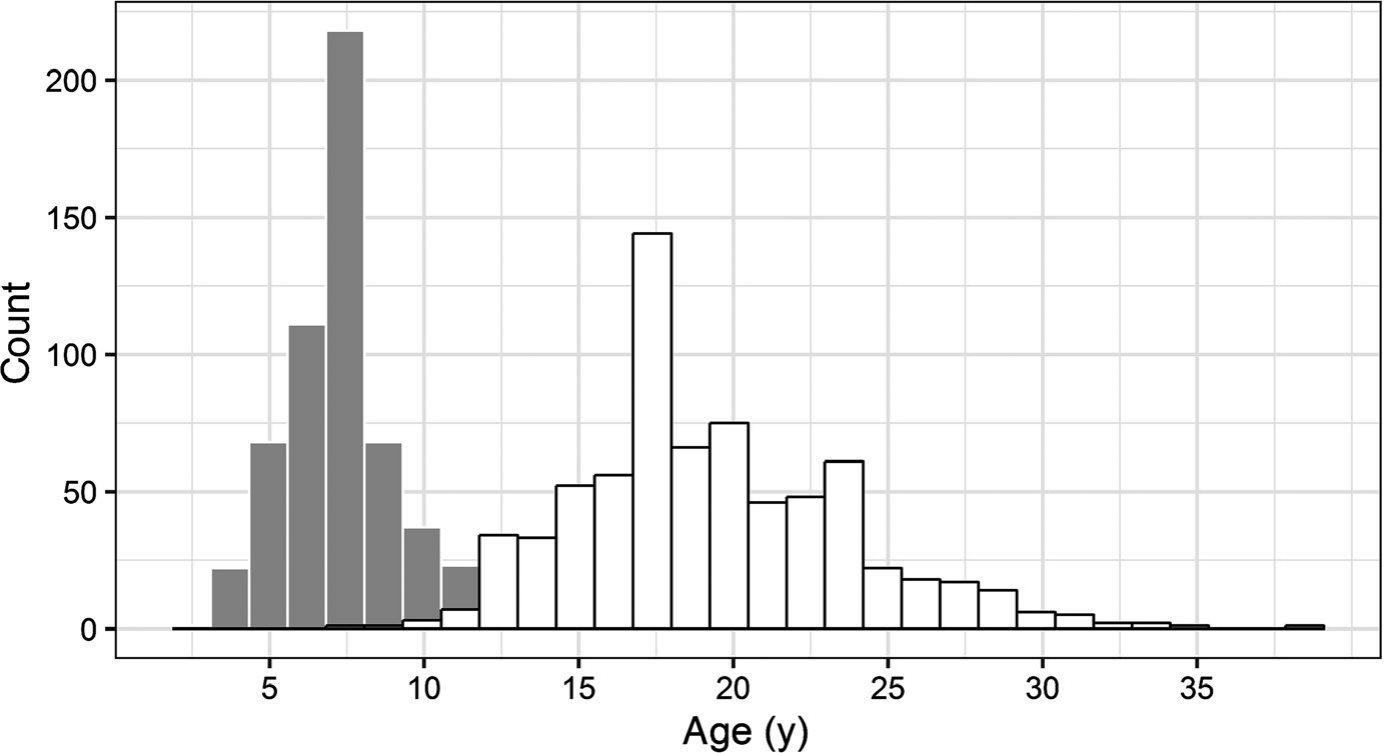
Age distribution of European eels (80s and 90s) from the river Imsa (Norway) based on otoliths read whole (“in toto” method: IT; gray bars) or grinded and polished (GP; white bars). The IT and GP age distributions were significantly different from each other (Kolmogorov–Smirnov, D = 0.925, *p* < .0001)

Estimated ages from the IT method were either equal or lower than from the GP method (Figure 5). Differences varied between 0 and 29 years with a mean and a median equal to 11 years. The correlation between age estimates from both methods was significant (*R* ^2^ = 0.05, *F*(1, 586) = 30.8, *p* < .0001), but still too low to infer one estimate from the other (Figure 5). The new and old age distributions were different from each other (Figure 6).

### 3.2 Comparison of new age estimates and length at silvering over the years

Out of the 798 eels, 751 were females, 43 males and four undifferentiated. Ninety‐three percent were at the silver stage, and the remaining were either yellow or intermediate.

**FIGURE 7:**
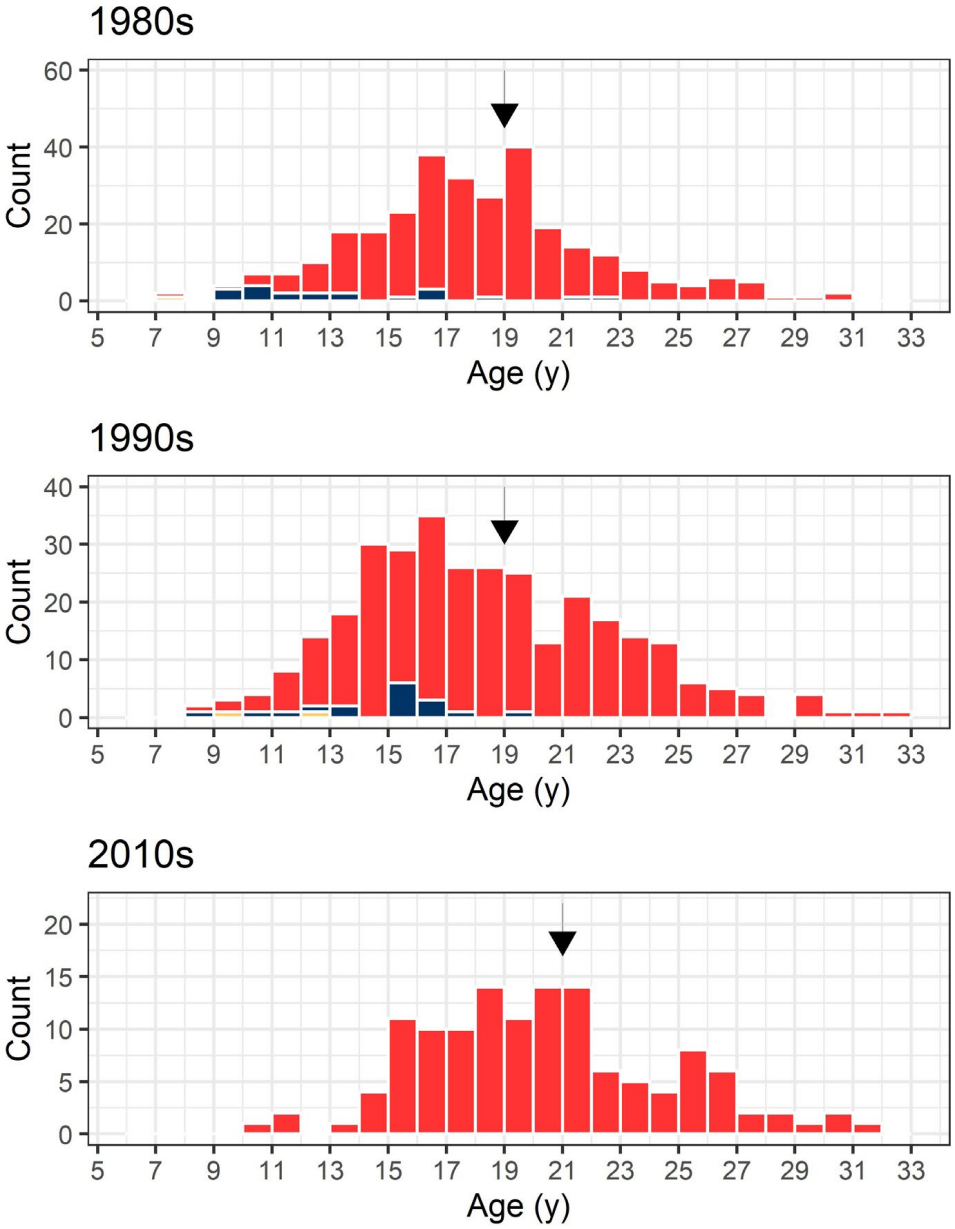
Age distribution of European eel (undifferentiated: yellow; males: blue, females: red) caught during their downstream migration in the river Imsa between 1982 and 2016. Mean (equal to median, shown as arrows) ages of females were 19 years in the 1980s and 1990s, and 21 years in the 2010s

Mean age at silvering (all years) was 19 years for females and 15 years for males (Table 1). During the last decade (2010s), mean age of females significantly increased compared to the 1980s and 1990s (Table 1). The same result was obtained when removing the OUI level 3 eels (Table 1). This is due to the disappearance of young silver eels (<15 years) during the 2010s (Figure 7). This corresponds to the lowest recruitment level which was reached in the 2000s (Figure 2), thus 12–15 years before the 2010s sampling.

**FIGURE 8:**
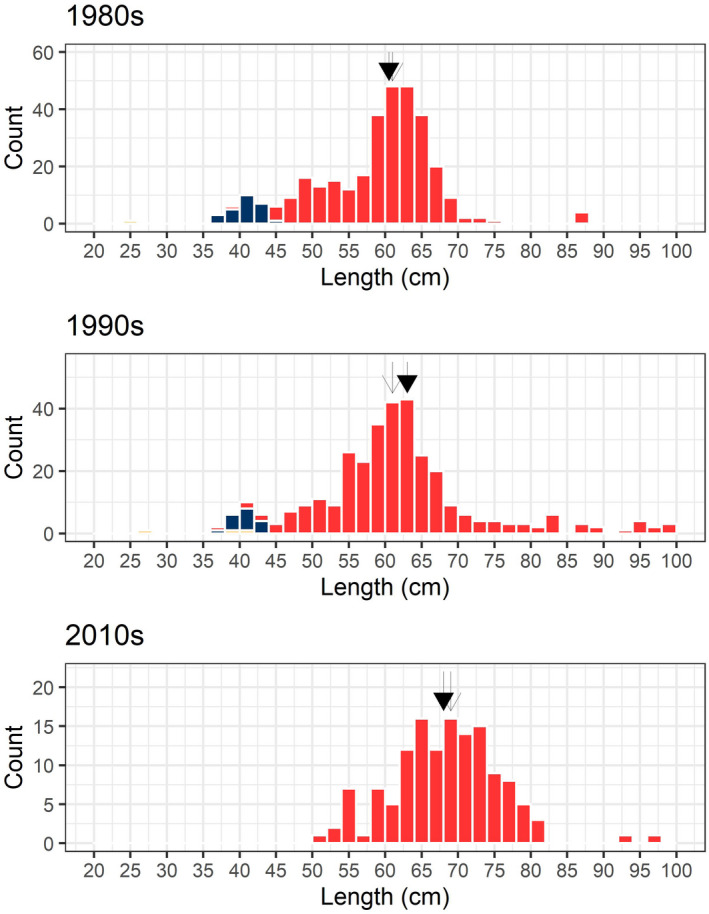
Length distribution of downstream migrating silver European eel (undifferentiated: yellow; males: blue, females: red) caught during their downstream migration in the river Imsa between 1982 and 2016. Mean length of females (closed arrow) was 60 cm in the 1980s, 63 cm in the 1990s, and 68 cm in the 2010s. Median lengths (open arrows) were 61 cm in the 1980s and 1990s and 69 cm in the 2010s

Mean body length increased significantly from the 1980s to the 2010s (Table 1, Figure 8). Female length increased by 8 cm over the 30‐year period (Table 1). When male silver eels were still caught in the trap, these migrated at a length of around 40 cm. In the 2010s, male eels disappeared, as did the contingent of smaller female silver eels (around 50 cm). We found no differences in length–weight relationship between decades (*F*(2, 241) = 1.37, *p* = .26) (Figure S1).

### 3.3 Growth

Growth estimated based on the GP method was highly variable, and age of female eels was only slightly related to their body length (*R* ^2^ = 0.08). Mean growth (Table 2) calculated over the entire freshwater stage of the eels by using the new age estimates (GP method) was 30 mm/year in females (min–max: 16–64 mm/year) and 24 mm/year in males (min–max: 15–37 mm/year). Mean growth calculated with the old estimates (IT method) was 77 mm/year for females and 72 mm/year for males.

**Table 2:** Summary of mean (min–max) growth (mm/year) of European eel from the river Imsa (Norway) calculated on age determined from otolith processed using two different methods “grinding and polishing” (GP) and “In toto” reading (IT)

Growth (mm/year)	GP method		IT method	
Decades	Females	Males	Females	Males
80s	30 (18–64)	26 (15–37)	72 (39–135)	72 (39–118)
90s	31 (16–60)	23 (17–36)	83 (42–142)	73 (37–114)
2010s	31 (17–57)	No data	No data	No data
All years	30 (16–64)	24 (15–37)	77 (39–142)	72 (37–114)

### 3.4 Linking annual numbers of recruits and silver eels

Both the number of ascending recruits (elvers) and descending silver eels have changed substantially during the period from 1975 to 2019 (Figures 2 and 3). The number of ascending recruits demonstrated large annual variation during the period 1975–1990, with a minimum of 3,000 and a maximum of 57,750 eels, and a mean number of 23,655 (±*SD* 19,039). From 1991 to 2019, the annual numbers of ascending recruits declined to a much lower level, varying between 5 and 11,078 eels, with a mean of 2,821 (±*SD* 2,950). The number of descending silver eels changed abruptly in 1988, from a mean of 3,888 (±*SD* 1 112) eels during 1975–1988 to 2,107 (±*SD* 789) during 1989–2019 (Figure 3).

The age distribution of the female silver eels migrating to the sea each year included up to 31 age classes (from 8 to 39 years old). Thus, each year's silver eel run represented more than 20 age classes of recruits. We attempted to fit a Recruit–Stock analysis model by assigning descending silver eels to recruit cohorts according to mean decadal age distributions but found no significant relationship.

## 4 DISCUSSION

Although widely geographically spread out, all European eels spawn in the Sargasso Sea and form one panmictic population (Als et al., 2011). Some of their biological characteristics, such as age at maturation, growth, and fecundity, can vary greatly depending on where they spend their growth phase, the yellow stage (Durif, Ginneken, Dufour, Müller, & Elie, 2009; Vøllestad, 1992). It is unknown whether eels from certain regions contribute more to the spawning stock and whether this may change from year to year. The success of *Anguilla anguilla* as a species is probably linked to its incredible plasticity in terms of life‐history strategies and biological characteristics. In the context of the decline, it is essential that all components of the population contribute to the spawning stock. The decline in recruitment has been more pronounced in the North than in the rest of Europe (1.9% vs. 8.9% of the references levels in 1960–1979, ICES, 2019). Norway represents the limit of the distribution area, and it is there that changes in densities are more likely be detected. The time series from the river Imsa is important for monitoring the stock. The Norwegian red list assessment for eel has also been based partly on this time series. Generation length is used to classify endangered species into the different IUCN categories. The previous Norwegian assessment has used a mean age at maturation of 8 years based on the previous studies (Vøllestad & Jonsson, 1986, 1988). The present study reporting a mean age of 19 years for female silver eels will likely have an impact on the next revision of the Norwegian red listing, by possibly re‐assigning the CR (critically endangered) or at the least EN (endangered) status to the European eel which is currently considered vulnerable (VU).

### 4.1 Otolith processing methods and reading uncertainty

As expected, there were large differences in age estimates of eels between the two different methods, in toto (IT) and grinding and polishing (GP). The age difference was 11 years on average with a maximum of 29 years. The differences were not proportional to age, but ages using GP were older than using IT. The present study confirms that GP is a better method for estimating age in the European eel than clearing of whole otoliths in ethanol. The cracking and burning method for reading eel otoliths has also been recommended, and it gives comparable results to GP (ICES, 2009). However, it has the disadvantage of being destructive. Burning the otolith will destroy marks left by chemical tagging. It is also not compatible with, for example, otolith chemistry analyses, which allow one to determine the salinity life history of eels. Cracking and burning was previously tested on otoliths of Imsa eels, but the burnt otoliths were difficult to read (Vøllestad & Jonsson, 1988).

Revealing annuli on the otoliths is not the only challenge related to age estimation in eels. A proper validation of age determination is still lacking, especially for older eels (over 20 years) from northern latitudes, where growth is slow. In other words, it is uncertain whether all the annuli represent winter marks, since some can be very tightly distributed, forming bundles of annuli. In the present study, it was considered unlikely that all these bundled marks represented an annual increment; rather, one year was assigned to each bundle. In the absence of definitive annulus identification, this was the best approach. The otolith of a 43‐year‐old eel kept in an aquarium for 22 years was recently analyzed (Palstra et al., 2020). The manner in which the annuli were counted was very similar to how we proceeded. This gives us extra confidence in our estimates since the age of this eel was known. If anything, our method of grouping certain annuli may have led to some under‐estimation, but this was to some degree accounted for in the Otolith Uncertainty Index (OUI).

Further, in our study, 5% of the otoliths were unreadable. In comparison, this proportion was 10%–30% for eels caught in Mediterranean lagoons where eels frequently change salinity and habitat (Panfili & Ximénès, 1994). Still, 39% of the otoliths from the Imsa were difficult to read (OUI, level 3: uncertainty >5 years). These may have qualified as “unreadable” by Panfili and Ximénès (1994), but here we chose to assign a high uncertainty rather than discarding them.

Using otoliths of known age, Svedäng et al. (1998) showed that younger eels were consistently over‐aged while older eels were under‐aged. The reason for overestimations was the presence of supernumerary zones in younger eels that were misidentified as annuli. For older eels, it is difficult to detect annuli in the outer slow‐growing part of the otolith. An additional inconsistency can be found in readings by the same reader over time which can amount to 6 years (Svedäng et al., 1998). Throughout *A. anguilla's* geographic range, the unknown age of glass eels at metamorphosis, depending on the location, may add one to two years of uncertainty to the total age. Similarly, the outer bands may not be fully revealed at the edge of the otolith by a polishing and grinding method causing an under‐aging.

Some otoliths, however, are very clear and can be easily interpreted. Therefore, it is important to include some measure of confidence around the age determination, at least, until there is a proper age validation method. We suggest a simple method by implementing an otolith uncertainty index (OUI) such as described in the present study. Changes in the thresholds between the OUI levels should, however, be adapted to the local eel sub‐population which is examined. For instance, an uncertainty of 3 years may not be ecologically or even statistically relevant for eels that live up to 30 years, but a 3‐year uncertainty will have more impact for younger, faster‐growing eels from the southern part of the distribution. Depending on the type of output where age data are needed, ranging from population dynamics models to management advice, subsets of data could be selected based on their OUI. However, OUI increases with age. In other words, otoliths of older eels are more difficult to interpret. Thus, removing subsamples of uncertain otoliths can bias the age structure. We recommend testing different configurations with different data subsets before making any conclusions. Finally, the development of machine learning methods for automatic otolith image analyses is promising (Moen et al., 2018). An OUI index will also be useful in that context, for selecting suitable learning datasets.

### 4.2 Evolution of the age distribution of silver eels in the river Imsa

As expected, age at silvering varied greatly in the eels from the river Imsa (females: 8–35 years; males: 9–23 years), but the overall mean varied only slightly across decades (from 19 to 21 years in the 2010s). Since most age readings had an associated uncertainty of 3 to 4 years, this 3‐year increase is meaningless, although statistically significant. Actually, given the disappearance of young silver eels (<15 years) during the more recent decades, it is surprising that the mean and median age were not more affected. However, mean age of silver eels is bound to increase even more in the river Imsa with the consistently low numbers of ascending recruits the last 2–3 decades. But in 2009 and 2014, elver recruitment increased and almost reached the 10,000‐individual threshold. An effect on the number of silver eels might not be detected before at least 10–15 years later. If these two peaks do affect the number of silver eels, it will not happen before 2022. In any case, if recruitment does not improve, the effect of these two years of increased recruitment will be short lived and perhaps nondetectable due to the low levels during most of the last 15 years.

### 4.3 Length at silvering

Eels are present in many types of habitats and salinities: coastal waters, lagoons, lakes, rivers, marshes, fjords, and estuaries. Length (and not only age) distributions can vary greatly among these habitats (Durif, Ginneken, et al., 2009; Holmgren, Wickström, & Clevestam, 1997; Melia et al., 2006; Poole et al., 2018; Svedäng et al., 1996; Vøllestad, 1992; Vøllestad & Jonsson, 1986). All eels need to accumulate fuel for the sustained high‐intensity swimming necessary for the journey to the Sargasso Sea, but females will face higher energetic demands in order to produce eggs. This leads to different life‐history strategies and a sexual dimorphism based on differences in length at maturity (Bertin, 1956; Tesch, 2003). Male eels migrate at around 35–45 cm (in this study 40 cm), minimizing the duration of their yellow stage, while females migrate at sizes of 40–130 cm, optimizing their size to reach a higher fecundity (Durif, Ginneken, et al., 2009; Helfman, Facey, & Hales, 1987; Tesch, 2003; Vøllestad, 1992). In the northern part of the distribution area, eels (males and females) are on average larger than in southern areas, and this has been linked to the increasing distance they have to swim to reach the spawning area (Durif, Ginneken, et al., 2009; Tesch, 2003; Vøllestad, 1992). Yet in the present study, which was located at a relatively high latitude (58.9°N), most female eels migrated at a body length around 60 cm and a small contingent of eels migrated at body length around 40–55 cm. Possibly, some eels could have stopovers on their way to the Sargasso Sea; but in the case of Norway, there is no obvious location for a stopover, since silver eel spawners take the northern route (north of the Shetlands) rather than through the Dover straight (Kettle, Vøllestad, & Wibig, 2011; Westerberg, Sjöberg, Lagenfelt, Aarestrup, & Righton, 2014). The best gonad‐to‐body size ratio under experimental artificial maturation was found in eels longer than 70 cm (Durif, Dufour, & Elie, 2006). Once a specific size is reached, a period of high growth probably triggers silvering (Durif et al., 2005; Huang et al., 1998). Recent work in reproductive endocrinology has identified the kisspeptin system as essential for the onset of puberty in mammals but also in teleost fish (Pasquier et al., 2018; Seminara et al., 2003; Zohar, Munoz‐Cueto, Elizur, & Kah, 2010). In eels, kisspeptins regulate the expression of gonadotropins. They may be the link between environmental factors and the reproductive axis through the regulation of growth hormone (Huang et al., 1998; Kim, Choi, Park, & Choi, 2015; Zohar et al., 2010).

### 4.4 Growth of eels

The new age estimates using the grinding and polishing (GP) method indicate that eels in the river Imsa spend a substantially longer period as yellow eels in freshwater than previously thought (Vøllestad & Jonsson, 1986). Previous estimates of silver eels in the river Imsa suggested a mean age of 5 years for male and 8 years for female silver eels (Vøllestad et al., 1986), while the new estimates indicated a mean age of 15 years for males and 19 years for females. At the time, it was concluded that eels in the river Imsa grew quickly, with a mean size increment of around 70 mm/year, which is comparable to growth in brackish water and in southern Europe (Acou et al., 2003; Rossi & Colombo, 1976; Vøllestad, 1985). In the river Imsa, Slower growth is more likely (this study: 30 mm/year), because at these latitudes the growth season is shorter than in southern Europe as eels stop feeding when the water is colder than 8–10°C (Riley, Walker, Bendall, & Ives, 2011; Vøllestad et al., 1986; Westerberg & Sjöberg, 2015). This was also visible through the patterns of the annuli. Tight, numerous rings are interpreted as short growth seasons. Our method to determine growth rate was simple and did not take into account changing growth rates over the lifetime. The new mean growth estimate in the river Imsa is 30 mm/year, which is less than half of what was previously documented. This new value is in line with newer growth estimates of eels in freshwater and in the northern part of the distribution range (Aprahamian, 2000; Arai, Kotake, & McCarthy, 2006; Lin, Ložys, Shiao, Iizuka, & Tzeng, 2007; Silm, Bernotas, Haldna, Järvalt, & Nõges, 2017; Simon, 2007, 2015).

Growth of eels in the river Imsa has not changed since the 1980s. This was contrary to what was expected. Water temperature has also increased due to climate change, and this has provided longer growth seasons. Additionally, although this has never been investigated, a reduced number of ascending recruits have led to a lower density of yellow eels in the freshwater habitat; this should have resulted in better growth and faster onset of the silvering process, leading up to silver eel descending at a younger age in recent years than in previous periods. Early analyses based on different aging methodology did indicate density‐dependent mortality in Imsa (Vøllestad & Jonsson, 1988), opening the possibility also for density‐dependent growth.

There were very few individuals younger than 15 years in the samples from the 2010s. This agrees with the large reduction in recruitment from the late 1990s. The recruitment has remained low since then, with almost no recruitment in several years in the mid‐2000s and later (Figure 2). This consistency gives us extra confidence in the new age estimations. Indeed, silver eels 15 years or less caught in the 2010s have entered the river after 1997–2001; hence, given the decline in recruitment, a large decline in this age group was expected. The IT method would have estimated most eels sampled in the 2010s to be around 10 years old with a cutoff value at 5 years, meaning a decline around 2007–2011. This was not the case, and therefore, estimates from the GP method are more likely.

### 4.5 Sex ratio

Male eels have always been scarce in the river Imsa; in the 1980s, they represented 3%–7% of the total run, but they all disappeared in the 2010s (Poole et al., 2018). Sex determination in eels is metagamic, meaning it is nongenetic (Geffroy & Bardonnet, 2016). Sex ratios are indeed skewed at individual localities, and there is a geographic bias associated with latitude and longitude (Davey & Jellyman, 2005; Helfman et al., 1987; Oliveira, McCleave, & Wippelhauser, 2001). The general pattern is that male eels are more abundant at southern latitudes and mainly in the lower reaches of rivers, whereas females dominate at higher latitudes and with increasing distance to the sea. Additionally, higher proportions of males are usually associated with high eel densities (Beentjes & Jellyman, 2003, 2015; Davey & Jellyman, 2005; Harrison, Walker, Pinder, Briand, & Aprahamian, 2014; Laffaille, Acou, Guillouët, Mounaix, & Legault, 2006; Parsons, Vickers, & Warden, 1977); although a study done in a laboratory showed opposite results (Huertas & Cerda, 2006). This later study, and others, also suggest that sex determination occurs during the first 3 months of growth (Davey & Jellyman, 2005; Huertas & Cerda, 2006).

The density factor may affect sex ratio through (a) food availability, depletion of food resources, and lower growth or (b) through social interactions: possibly through odors of conspecifics or even through cannibalistic behaviors which would skew the sex ratio since females are larger than males (Davey & Jellyman, 2005). Eel density in the Imsa catchment has severely decreased following the decline in recruitment since the late 2000s (Figure 2). Future work including back‐calculation of length at age on these otoliths could bring some insight into whether growth after freshwater recruitment has changed since the 1980s.

### 4.6 Link between ascending recruits and descending silver eels

Our attempts to calculate mortality relied on too many assumptions to give a reliable value and were therefore discarded. However, given the low annual number of ascending juvenile eels and the relatively high number of silver eels, a mean age of silver eels at 19 years for females and 15 years for males suggests that mean annual mortality in freshwater has to be very low («10%). The termination of eel fishing in the Imsa water course in 2006 has likely contributed to a reduced freshwater mortality, but natural mortality may anyway appear to have been very low all through the study period. In a river–lake system mainly inhabited by invertebrate‐feeding brown trout (*Salmo trutta*), whitefish (*Coregonus lavaretus*), Arctic charr (*Salvelinus alpinus*), and three‐spined sticklebacks (*Gasterosteus aculeatus*), the main predation mortality in eels is likely restricted to the very early yellow eel stages. There are reports of minks being caught in the trap and which probably also cause some mortality. Cormorants also likely induce some mortality, but for which there is no information.

Because of the long residency in freshwater (>15 years) for eels in the river Imsa, even a time series of more than 40 years is too short to allow a robust analysis of the relationship between the number of ascending recruits and the resultant number of descending silver eels. The wide silver eel age distributions, together with the stochastic environmental effect on the silvering process and annual number of descending eels, mask any potential signal from the variation in number of recruits. The annual variation in the number of recruits will be reflected in a large number of silver eel cohorts, resulting in a very smoothed signal from the variation in recruits. For example, as the age variation in female silver eels in river Imsa spans 35 years (minimum age = 5 years and maximum age = 39 years), a time series of 44 years (since 1975) will only include the complete number of silver eels for approximately five cohorts of recruits. In addition, as the environmental and habitat variables may have changed substantially during the 44 year period (for example temperature, Poole et al., 2018), we cannot expect a stable relationship between the numbers of ascending recruits and silver eels over the years, and attempting to split the time series into periods with relatively similar environmental conditions and fit models to these will be futile.

In addition, we know little about the factors governing growth, mortality, and strategic choices during the freshwater life phase of eels, so it will be difficult to parameterize a model adequately. For example, how should we include the effect of density in the model? Will reduced overall densities mainly affect densities in unfavorable habitats or habitats further from the sea (above the lakes), while density remains high in favorable habitats, as indicated by Boulenger et al. (2016)? Will the effect of increased density be increased mortality, due to more competition for resources or more predation from older eels, or reduced growth due to displacement to lower quality habitats? At very low densities, other effects like Allee effects, depensatory mechanisms, changing sex ratios or life‐history strategies can also obscure the relationship (see references in Poole et al., 2018; Sandlund et al., 2017). One should also note that developing river‐wise stock‐recruitment models for European eel is not possible. The species is panmictic (Als et al., 2011; Palm, Dannewitz, Prestegaard, & Wickström, 2009), with a biology that implies a weak, or no, connection between the number of silver eels leaving any watercourse for spawning in the Sargasso Sea and the number of glass eels returning to that watercourse.

In conclusion, the method of revealing annuli is one of the elements that can improve the precision and the accuracy of age estimates. Grinding and polishing the otolith seems a better method than reading the age “in toto” for older eels with a lifetime of more than one decade. However, beyond the method, there are two types of errors associated with age determination in fish: A process error related to how well the otolith reflects the complete growth record of the fish throughout its lifetime, and observation errors linked to the interpretation of these annuli (Campana, 2001). In eels, several studies have verified the correspondence between otolith structures and seasonal increments (Chrisnall & Kalish 1993; Moriarty, 1983; Oliveira, 1996; Svedäng et al., 1998); however, reading age of slow‐growing eels remains a challenge. Separating false checks from real winter marks will require a proper validation of the growth increments, especially for the northern part of the distribution area where growth is slower and occurs over a shorter period per year. The new‐age distribution we determined, however, was consistent with the dynamics of elver recruitment in the river Imsa since 1975. This gives us some extra confidence in our age determination: Eels have been spending on average 19 years in freshwater since the 1980s, and this has only slightly increased during the 2010 (mean of 21 years). Still, the variation around these numbers is considerable, from 5 to 39 years, and this means that eels from up to 34 cohorts of recruits (elvers, small yellow eels) can be included in each year's group of descending silver eels. In this case, developing a model that links annual numbers of ascending recruits and silver eels is likely futile.

## CONFLICT OF INTEREST

None declared.

## AUTHOR CONTRIBUTION

**Caroline Durif:** Conceptualization (lead); Data curation (equal); Formal analysis (lead); Investigation (lead); Methodology (lead); Project administration (equal); Visualization (lead); Writing‐original draft (lead); Writing‐review & editing (lead). **Ola Håvard Diserud:** Conceptualization (equal); Formal analysis (equal); Investigation (equal); Methodology (equal); Writing‐original draft (equal); Writing‐review & editing (equal). **Odd Terje Sandlund:** Conceptualization (equal); Funding acquisition (equal); Project administration (equal); Writing‐review & editing (equal). **Eva Thorstad:** Conceptualization (equal); Data curation (equal); Funding acquisition (lead); Project administration (equal); Writing‐review & editing (equal). **Russell Poole:** Conceptualization (equal); Investigation (equal); Methodology (equal); Writing‐review & editing (equal). **Knut Bergesen:** Data curation (equal); Methodology (equal); Resources (equal); Writing‐review & editing (equal). **Rosa Helena Escobar‐Lux:** Data curation (equal); Formal analysis (equal); Investigation (equal); Methodology (equal); Writing‐review & editing (equal). **Steven Shema:** Data curation (equal); Formal analysis (equal); Investigation (equal); Methodology (equal); Writing‐review & editing (equal). **Leif Asbjørn Vøllestad:** Conceptualization (equal); Data curation (equal); Formal analysis (equal); Investigation (equal); Methodology (equal); Writing‐review & editing (equal).

## Supporting information

Figure S1

## Data Availability

Data are available in Dryad, accession number: https://doi.org/10.5061/dryad.2jm63xskk.
