# Supplementary figures and images for: Age of European silver eels during a period of declining abundance in Norway
Source: Ecol Evol. 2020 Apr 12;10(11):4801–15. doi: 10.1002/ece3.6234 (PMC7297751; doi:10.1002/ece3.6234)

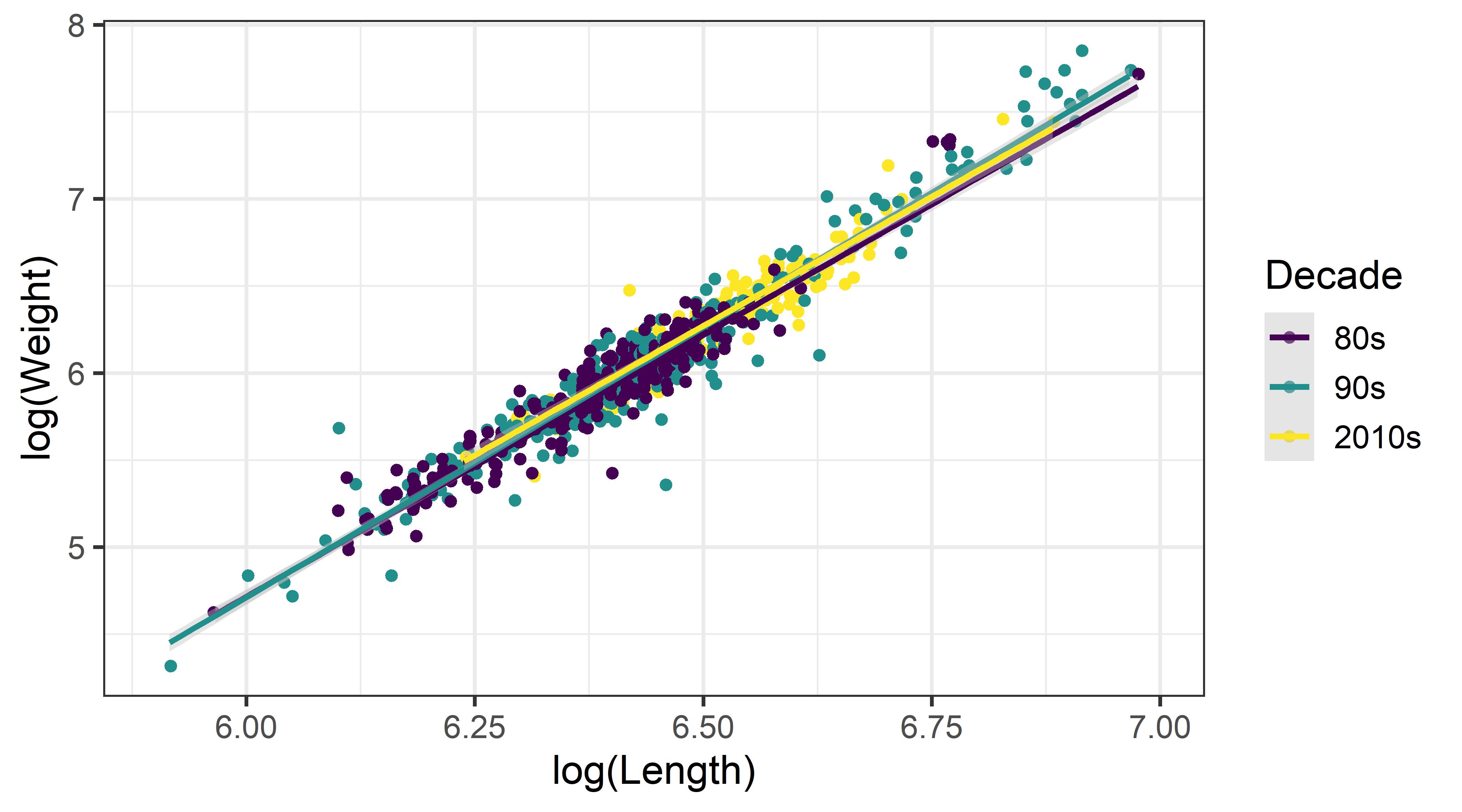

Supplement: Supplementary file 1 — Figure S1 [file ECE3-10-4801-s001.jpg]
